# Integrative taxonomy of root aphid parasitoids from the genus *Paralipsis* (Hymenoptera, Braconidae, Aphidiinae) with description of new species

**DOI:** 10.3897/zookeys.831.31808

**Published:** 2019-03-18

**Authors:** Milana Mitrović, Petr Starý, Miljana Jakovljević, Andjeljko Petrović, Vladimir Žikić, Nicolás Pérez Hidalgo, Željko Tomanović

**Affiliations:** 1 Institute for Plant Protection and Environment, Department of Plant Pests, Banatska 33, Belgrade, Serbia Institute for Plant Protection and Environment Belgrade Serbia; 2 Laboratory of Aphidology, Institute of Entomology, Biology Centre of the Czech Academy of Sciences, Branišovská 31, 37005 České Budějovice, Czech Republic Biology Centre of the Czech Academy of Sciences České Budějovicé Czech Republic; 3 University of Belgrade-Faculty of Biology, Institute of Zoology, Studenstki trg 16, 11000 Belgrade, Serbia University of Belgrade Belgrade Serbia; 4 University of Niš-Faculty of Science and Mathematics, Department of Biology and Ecology, Višegradska 33, Niš, Serbia University of Niš Niš Serbia; 5 Institut de Biologia Integrativa de Sistemes (I2SysBio) Universitat de València-CSIC, C/. Catedràtic Agustín Escardino Benlloch, 46908 Paterna, Valencia, Spain Universitat de València València Spain

**Keywords:** Cryptic speciation, molecular phylogeny, *
Paralipsis
*, *Paralipsisbrachycaudi* sp. n., *Paralipsisrugosa* sp. n.

## Abstract

Species from the genus *Paralipsis* are obligatory endoparasitoids of root aphids in the Palaearctic. It is known that these species are broadly distributed, parasitizing various aphid hosts and showing great biological and ecological diversity. On the other hand, this group of endoparasitoids is understudied and was thought to be represented by a single species in Europe, viz., *Paralipsisenervis* (Nees). However, recent description of two new species indicated the possibility of cryptic speciation and recognition of additional *Paralipsis* species in Europe. In this research, *Paralipsis* specimens collected during the last 60 years from eight European countries, as well as one sample from Morocco, were subjected to molecular and morphological characterization. Newly designed genus-specific degenerative primers successfully targeted short overlapping fragments of COI of the mitochondrial DNA. Molecular analyses showed clear separation of four independent lineages, two of which are the known species *P.enervis* and *P.tibiator*, while two new species are described here, viz., *P.brachycaudi* Tomanović & Starý, **sp. n.** and *P.rugosa* Tomanović & Starý, **sp. n.** No clear specialization of the taxa to a strict root aphid host has been determined. The recognized mitochondrial lineages were distinct one from another, but with a substantial within-lineage divergence rate, clearly indicating the complexity of this group of parasitoids, on which further research is required in order to clarify the factors triggering their genetic differentiation. We reviewed literature data and new records of *Paralipsisenervis* aphid host associations and distributions. A key for the identification of all known *Paralipsis* species is provided and illustrated.

## Introduction

**Figure 1.**
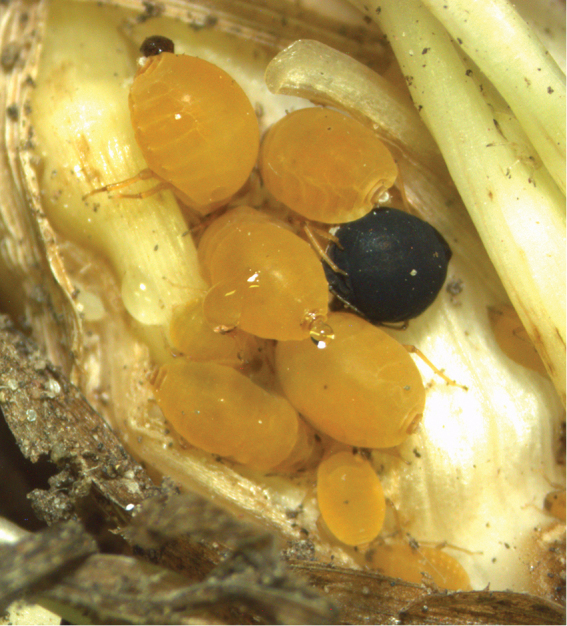
*Forda* sp. aphids colony on root *Dactylis* sp. with mummy of *P.enervis*.

Parasitoid wasps from the subfamily Aphidiinae (Hymenoptera, Braconidae) attack various aphid phylogenetic lineages, exhibiting several specialized associations with their hosts (Gagić et al. 2016). Few parasitoid wasps are specialized to parasitize only root aphid species (Starý 1961). However, there is no substantial biological knowledge about these obligatory parasitoid species of root aphids, probably as a consequence of them being of little economic importance and difficult to access for sampling. Moreover, it is well known that the parasitoids of root aphids developed obligatory relationships with ants (Starý 1966, Takada and Shiga 1974, Völkl 1992, Völkl et al. 1996). Although, there are many examples of relationships between ants that collected honeydew from aphids and protected the aphid colony and parasitoid wasps, it seems that chemical mimicry plays a more important role in some parasitoids [e.g., *Lysiphlebuscardui* (Marshall 1896)] than behavioural mimicry (Liepert and Dettner 1993). However, parasitoid wasps from the genus *Paralipsis* Foerster, 1863 have developed species-specific relationships with ants attending root aphids (Starý 1966, Takada and Shiga 1974, van Achterberg and Ortiz de Zugasti Carrón 2016). The genus *Paralipsis* is а good example as case of specific obligatory parasitoids of root aphids in Europe and the Palaearctic (Figure 1). Until recently, *Paralipsisenervis* (Nees) was considered to be the only European species, while *P.eikoae* (Yasumatsu) was known as a Far Eastern species (Starý and Schlinger 1967). However, after examining two samples of *Paralipsis* from Spain and the Netherlands, van Achterberg & Ortiz de Zugasti Carrón (2016) described two new species on the basis of morphological characters, viz., *P.planus* van Achterberg and *P.tibiator* van Achterberg and Ortiz de Zugasti. It is known that the genus *Paralipsis* shows great biological and ecological complexity and diversity in view of their acceptance of various aphid hosts and also having a broad geographical distribution.

Bearing in mind it was thought that the genus *Paralipsis* was represented by a single species in Europe, until additional two species were newly described recently, we started this research to address the possibility of cryptic speciation and recognition of additional *Paralipsis* species in Europe. Since rarely encountered, there is almost a complete lack of knowledge about morphology and reliable characters for diagnostics of these root aphid parasitoids.

A set of wasps collected during the last 60 years from eight European countries and Morocco were initially subjected to morphological characterization. In addition, DNA was extracted from available *Paralipsis* specimens to perform the amplification and sequencing of the mitochondrial DNA barcoding region of cytochrome c oxidase subunit I (COI). We developed DNA amplification protocol and designed new internal genus-specific degenerative primers in order to retrieve short overlapping COI fragments for molecular characterization of the wasps. Subsequently, we used an integrative approach analyzing the morphological and molecular results to recognize phylogenetic lineages and cryptic species within the analyzed *Paralipsis* specimens. Two new species in Europe were described. In addition, we reviewed the host aphids and distribution of associations for *Paralipsisenervis*. A new determination key including all previously known and two newly described species is provided and illustrated.

## Materials and methods

### Insect material

**Figure 2.**
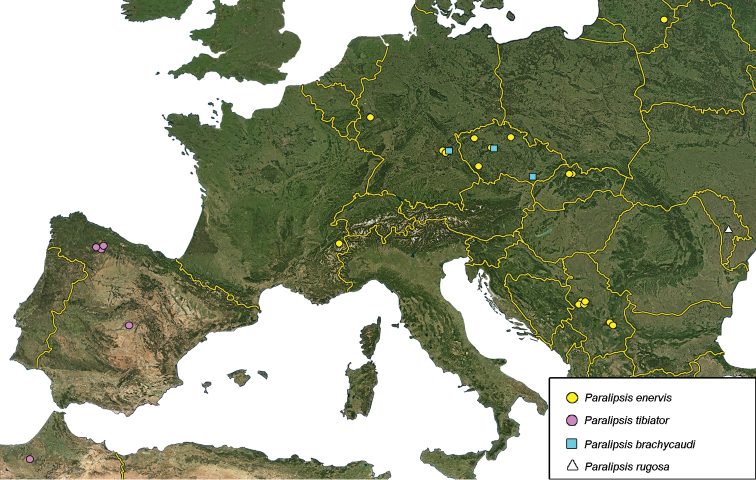
Distribution of analyzed *Paralipsis* specimens in Europe including Morocco.

We were provided with *Paralipsis* specimens collected during the last 60 years from eight European countries (Czech Republic, France, Germany, Lithuania, Moldova, Serbia, Slovakia, and Spain), in addition to one non-European sample from Morocco (Figure 2). Material was obtained by rearing from 17 different plant/aphid trophic associations, which included specimens emerging from 13 different aphid hosts (Table 1). Additionally, the paratype of *P.planus* was provided by the Naturalis Biodiversity Center, Leiden, the Netherlands.

**Table 1.** The list of available *Paralipsis* specimens subjected to molecular analyses.

Code*	Aphid host	Plant	Sampling year /age of sample at the time of DNA extraction	Sampling locality, collector	Country
Pr1Rd*	*Forda* sp.	*Dactylisglomerata* L.	2016 / 2	Niš, Sićevačka klisura, lgt. V Žikić	Serbia
PA1	*Anoecia* sp.	*Agropyrum* sp.	1960 / 58	Hořenec, BM 60/706, lgt. P Starý	Czech Republic
PA2	*Anuraphisfarfarae* (Koch)	*Tussilagofarfara* L.	1969 / 49	Leverkusen, Rheinland, lgt. M Boness	Germany
PA3	*Brachycaudusballotae* (Passerini)	*Ballotanigra* L.	1960 / 58	Praha, lgt. J Holman	Czech Republic
PA4	* Anuraphis farfarae *	* Tussilago farfara *	1974 / 44	Stankovany, Choc pohorie, lgt. P Starý	Slovakia
PA5	*Dysaphiscrataegi* (Kaltenbach)	*Daucuscarota* L.	1959 / 59	Praha, lgt. Pintera	Czech Republic
PA6	*Fordamarginata* Koch	*Agropyronrepens* L.	No data	Erlangen, Nordbayern, lgt. H Zwolfer	Germany
PA7	*Aphislambersi* (Börner)	*Daucuscarota* L.	1974 / 44	Stankovany, Choc pohorie, lgt. P Starý	Slovakia
PA8	*Aphis* sp.	*Potentillaanserina* (L.)	1963 / 55	Sušice, B m, lgt. J Holman	Czech Republic
PA9	Unknown	*Pastinacasativa* L.	1959 / 59	Jičín, Bor, lgt. J Holman	Czech Republic
PA10	*Fordaformicaria* von Heyden	*Poapratensis* L.	No data	Erlangen, Nordbayern, lgt. H Zwolfer	Germany
PA11	*Brachycaudusmordvilkoi* Hille Ris Lambers	*Echiumvulgare* L.	No data	Čejč, Mm, lgt. J Holman	Czech Republic
PA12	Unknown	Unknown	1960 / 58	Kisinev, lgt. Adaškevič	Moldova
PA13	*Tetraneuraulmi* (L.)	*Avenasativa* L.	No data	Erlangen, Nordbayern, lgt. H Zwolfer	Germany
PA14	*Dysaphisreaumuri* (Mordvilko)	*Ranunculus* sp.	No data	Le Combe, Passy, Ht Savoie, lgt. G Remaudiere	France
PA15	*Aphisrumicis* L.	*Rumex* sp.	1987 / 31	Immezeur, lgt. Sekkar	Morocco
PA16	* Forda marginata *	*Poaannua* L.	No data	Molety, raj, lgt. Zickai	Lithuania
PA17	* Forda formicaria *	Poaceae	2013 / 5	Morales del Arcediano, Leon, lgt.N Pérez Hidalgo	Spain
PA18	* Forda formicaria *	Poaceae	2013 / 5	Morales del Arcediano, Leon, lgt. N Pérez Hidalgo	Spain
PA19	* Forda formicaria *	Poaceae	2013 / 5	Morales del Arcediano, Leon, lgt. N Pérez Hidalgo	Spain
PA20*	* Forda formicaria *	*Setariaviridis* L.	1996 / 22	Sićevačka klisura, lgt. V Žikić	Serbia
PA21	* Forda formicaria *	*Bromussterilis* L.	1998 / 20	Petnica, lgt. Ž Tomanović	Serbia
PA22	* Forda formicaria *	* Bromus sterilis *	1998 / 20	Petnica, lgt. Ž Tomanović	Serbia
PA23	* Forda formicaria *	* Bromus sterilis *	1998 / 20	Petnica, lgt. Ž Tomanović	Serbia
PA24	* Forda formicaria *	* Bromus sterilis *	1998 / 20	Petnica, lgt. Ž Tomanović	Serbia
PA26*	* Forda formicaria *	Unknown	2015 / 3	Madrid	Spain

*specimens preserved in 96% ethanol prior to DNA extraction, while the others were stored dry in the collections.

Our examination of *Paralipsis* specimens took into account reliable morphological characters used in aphidiinae taxonomy (number of flagellomeres, shape of flagellomere 1 and 2, number of labial and maxillary palpomeres, size and shape of fore tarsus, shape of hind tibia and femur, wing venation pattern, pterostigma shape, ratio between the pterostigma and radial vein 1, petiole shape, propodeal areolation, and ovipositor shape) (Kavallieratos et al. 2005, van Achterberg and Ortiz de Zugasti Carrón 2016, Tomanović et al. 2018). The morphological terminology used in this article for diagnostic characters of aphidiines is based on Sharkey and Wharton (1997).

### DNA extraction, PCR, and sequencing

The barcoding region of the mitochondrial cytochrome oxidase c subunit I gene (COI) was chosen for phylogenetic study as a proven informative marker in species delineation for numerous aphidiines (Derocles et al. 2012, Tomanović et al. 2018). Most of the samples subjected to molecular analyses were dry and stored in entomological collections (Biology Center, Institute of Entomology, České Budějovice, Czech Republic [abbreviation IECR] – specimens from Czech Republic, Germany, Slovakia, Moldova, France, Morocco, Lithuania; Faculty of Biology, Institute of Zoology, Belgrade, Serbia [abbreviation FBS] - specimens from Serbia and Czech Republic) prior to DNA extraction, except for several samples that were kept in 96% alcohol (Table 1).

DNA extraction was conducted using a commercial DNeasy Blood and Tissue Kit (Qiagen Inc., Valencia, California, USA) following the manufacturer’s instructions. Initially, we attempted to amplify the barcoding region of the COI gene from dry material using the standard primer pair LCO1490/HCO2198 (Folmer et al. 1994). Each reaction was carried out in a volume of 20 μl, according to the following protocol: i) initial denaturation 95 °C/5 min; ii) 35 cycles including three steps, viz., 1 min/94 °C, 1 min/54 °C, and 30 sec/72 °C; and iii) final extension at 72 °C for 7 min.

**Figure 3.**
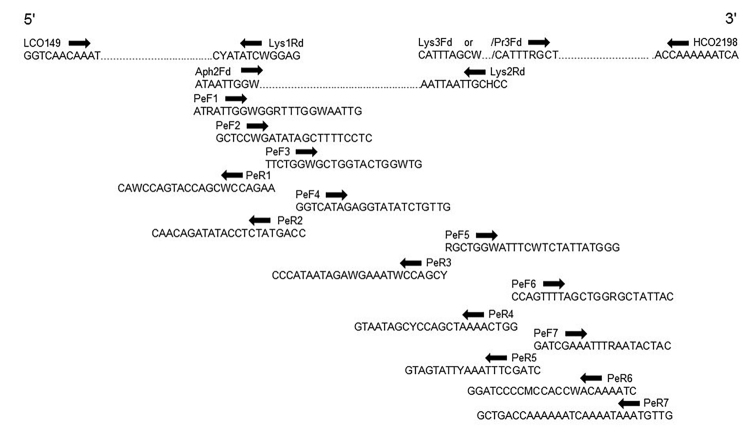
Distribution of the primers used in retrieving short overlapping barcode fragments of COI from dry *Paralipsis* specimens.

Since the standard primer pair failed to successfully amplify the barcoding region in more than three specimens, the next step was to test the suitability of the internal degenerative primers designed by Mitrović and Tomanović (2018) for dry museum specimens of other Aphidiinae genera. Partial success was achieved in such trials amplifying random fragments, but predominantly in the first 200–350 base pairs (bp) of the barcoding region, imposing the need to design new *Paralipsis*-specific primers with the aim to target the middle and last portions of mitochondrial DNA fragments. In the absence of reference COI sequences of *Paralipsis* parasitoids in the available public databases, we used our own sequences to design internal primers for dry material. These primers were positioned to amplify the missing fragments of COI, which could later be concatenated to longer barcoding sequences (Figure 3). Trials of retrieving the COI barcodes included PCR reactions combining the standard primers LCO1490 and HCO2198, ones designed by Mitrović and Tomanović (2018), and newly designed *Paralipsis*-specific primers, targeting overlapping fragments of different lengths and positions (Table 2, Figure 3).

**Table 2.** The list of primers used for retrieval of COI sequences from dry Paralipsis specimens.

**primer name**	**5 ’ 3’ primer sequence**	**primer direction**	**Reference**
LCO1490	GGTCAACAAATCATAAAGATATTGG	Forward	Folmer et al. (1994)
HCO2198	TAAACTTCAGGCTGACCAAAAAATCA	Reverse	
Aph2Fd	ATAATTGGWGGATTTGGWAATTG	Forward	Mitrović and Tomanović (2018)
Lys1Rd	GAGGAAAAGCYATATCWGGAG	Reverse	
Lys2Rd	GTWCTAATAAAATTAATTGCHCC	Reverse	
Lys3Fd	CATTTAGCWGGDATTTCWTC	Forward	
Pr3Fd	CATTTRGCTGGWATTTCYTC	Forward	
PeF1	ATRATTGGWGGRTTTGGWAATTG	Forward	*Paralipsis*-specific newly designed primers
PeF2	GCTCCWGATATAGCTTTTCCTC	Forward	
PeF3	TTCTGGWGCTGGTACTGGWTG	Forward	
PeR1	CAWCCAGTACCAGCWCCAGAA	Reverse	
PeF4	GGTCATAGAGGTATATCTGTTG	Forward	
PeR2	CAACAGATATACCTCTATGACC	Reverse	
PeF5	RGCTGGWATTTCWTCTATTATGGG	Forward	
PeR3	CCCATAATAGAWGAAATWCCAGCY	Reverse	
PeF6	CCAGTTTTAGCTGGRGCTATTAC	Forward	
PeR4	GTAATAGCYCCAGCTAAAACTGG	Reverse	
PeF7	GATCGAAATTTRAATACTAC	Forward	
PeR5	GTAGTATTYAAATTTCGATC	Reverse	
PeR6	GGATCCCCMCCACCWACAAAATC	Reverse	
PeR7	GCTGACCAAAAAATCAAAATAAATGTTG	Reverse	

Products of PCR were obtained in 40 μl following the protocol described by Mitrović and Tomanović (2018). All barcoding products were sequenced with forward and reverse primers for each part of the barcoding region using automated sequencing equipment (Macrogen Inc, Seoul, South Korea). Short barcode fragments were manually edited in FinchTV ver. 1.4.0 (www.geospiza.com), aligned and concatenated using the Clustal*W* program integrated in MEGA5 (Tamura et al. 2011). Sequenced mitochondrial barcodes were subjected to maximum likelihood best fit model analysis using the MEGA5 program. According to the obtained Akaike information criterion scores, the best fit model to calculate evolutionary distances was the Tamura-Nei model (Tamura and Nei 1993).

Maximum likelihood (ML) and maximum parsimony (MP) trees were constructed using the MEGA5 software, with 500 bootstrap replicates performed to assess the branch support (Felsenstein 1985). Another parasitoid belonging to the same subfamily (Aphidiinae), *Aphidiussussi* Pennachio and Tremblay, 1989, was used as an outgroup. A median-joining network (Bandelt et al. 1999) using maximum parsimony calculation was constructed with the NETWORK ver. 4.6.1.2 (http://www.fluxus-engineering.com).

## Results

**Figure 4.**
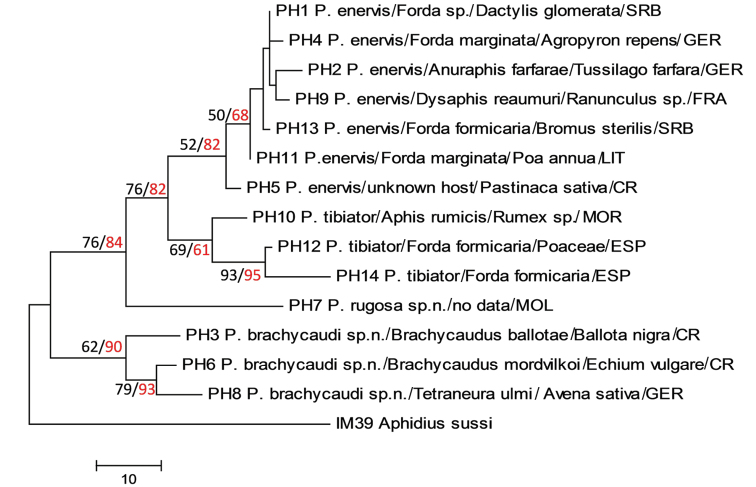
Phylogenetic relationship inferred using the maximum parsimony (MP) method. The consistency index is (0.533333), the retention index is (0.681818), and the composite index is 0.476540 (0.363636) for all sites and parsimony-informative sites. The MP tree was obtained using the subtree-pruning-regrafting (SPR) algorithm with search level 1, in which the initial trees were obtained by the random addition of sequences (10 replicates). The percentage of replicate trees in which >50% of the associated taxa clustered together in the bootstrap test (500 replicates) is shown next to the branches (in red color). Since the topology is identical, the bootstrap support of branches obtained by the maximum likelihood method is presented in black color as well. Barcoding haplotypes of the analyzed archival *Paralipsis* specimens are designated with codes from PH1 to PH14, species name, host aphid and host plant. Abbreviations for the countries of origin are as follows: **GER** – Germany; **FRA** – France; **SRB** – Serbia; **LIT** – Lithuania; **CR** – Czech Republic; **MOL** – Moldova; **MOR** – Morocco; and **ESP** – Spain.

Barcoding fragments of COI were successfully recovered from 18 specimens. The material subjected to molecular analyses was of different ages in terms of the time passing between sampling until DNA extraction; several of the oldest had been preserved in collections for nearly 60 years. This probably caused DNA disintegration, which resulted in failed attempts to recover the barcoding region with the LCO1490/HCO2198 standard primer pair. The newly designed *Paralipsis*-specific primers made it possible through diverse combinations to retrieve short subsequences of different length and position from disintegrated DNA of archival specimens. Prior to molecular analyses, all the barcoding sequences were aligned and trimmed to the same length of 568 bp. Comparison of COI barcodes identified 14 haplotypes (PH1-PH14) distinguished by a total of 83 variable sites, of which 51 were parsimony-informative (Table 3). The phylogenetic relationship was inferred using the MP and ML methods, which resulted in trees sharing identical topology with no substantial differences in bootstrap support (Figure 4).

**Table 3.** The list of identified barcoding COI haplotypes in the analyzed *Paralipsis* specimens.

**Haplotype**	**Specimens sharing the haplotype**	**Accession number of haplotype in GenBank**
PH1	Pr1	MH475319
PH2	PA2	MH475320
PH3	PA3	MH475321
PH4	PA6	MH475322
PH5	PA9	MH475323
PH6	PA11	MH475324
PH7	PA12	MH475325
PH8	PA13	MH475326
PH9	PA4	MH475327
PH10	PA15	MH475328
PH11	PA16	MH475329
PH12	PA17, PA18, PA19	MH475330
PH13	PA21, PA23, PA24	MH475331
PH14	PA26	MH475332

Phylogenetic analysis showed molecular differentiation on the basis of COI barcoding fragments, with recognition of four distinct lineages. The first group includes seven haplotypes: PH1, PH2, PH4, PH5, PH9, PH11, and PH13, which morphologically correspond to the first known species in this genus and in Europe, *P.enervis*. The specimens were sampled from different aphid hosts (*Forda*, *Aphis*, *Anuraphis*, *Dysaphis*) in association with different plants originating from Serbia, Germany, France, Lithuania, and the Czech Republic. The average overall divergence rate between the haplotypes within this group was 1%, with distances ranging from 0.4 to 2.5% (Table 4).

The second lineage, a “Mediterranean” clade, includes haplotypes PH12 and PH14 from Spain, and haplotype PH10 from Morocco. The overall divergence rate within this group was 2.8%. Genetic distances show that the haplotype PH12 associated with *Fordaformicaria* is intermediary, diverging from the haplotype PH10 from *Aphisrumicis* (2.4%) and from the haplotype PH14 associated with *Fordaformicaria* (2%), while the genetic distance between the other two was 4% (Table 4). Haplotype PH14 belongs to the paratype specimen of the newly described species *P.tibiator*. On the basis of morphological examination, it can be concluded that the haplotypes PH12 and PH10 belong to *P.tibiator*, although with evident high intraspecific genetic diversity. These three specimens clearly differ from the other congeners in having an elongated flagellomere 1 (F_1_) (the ratio between F_1_ and F_2_ is 1.3–1.4) and a large number of longitudinal placodes on F_1_ and F_2_ in males (5–6 in *P.tibiator* versus 0–2 in other *Paralipsis*).

The third distinct lineage on the phylogenetic tree consists solely of the haplotype PH7, with unknown host data. The single specimen available from Moldova is characterized by having a very rugose and irregularly carinated propodeum. It is described as the new species *P.rugosa* sp. n., clearly separated genetically, with average distance from the first, second, and fourth lineage of 7.3, 7.7, and 9.6%, respectively.

Three haplotypes (PH3, PH6, and PH8) originating from *Brachycaudus* sp. and *Tetraneuraulmi* aphid hosts from Central Europe (Czech Republic and Germany) are grouped within the fourth distinct lineage. The barcoding haplotypes differ in the range of 1.8 to 3.6%, with an average overall interlineage divergence rate of 2.8% (Table 4). Specimens of *Paralipsis* within this lineage are characterized by having a more elongated petiole and ovipositor sheath in comparison with other congeners, and are described as the new species *P.brachycaudi* sp. n.

**Table 4.** Genetic distances between the COI barcoding haplotypes of *Paralipsis* calculated using the Tamura-Nei method.

**Group**	**Haplotype**	**Tamura-Nei evolutionary distances**												
1	PH1													
	PH2	0.009												
	PH4	0.004	0.013											
	PH5	0.016	0.025	0.016										
	PH9	0.005	0.011	0.009	0.022									
	PH11	0.005	0.014	0.005	0.011	0.011								
	PH13	0.004	0.013	0.007	0.016	0.009	0.005							
2	PH10	0.044	0.046	0.044	0.036	0.050	0.042	0.048						
	PH12	0.050	0.056	0.050	0.037	0.056	0.048	0.054	0.024					
	PH14	0.063	0.070	0.059	0.050	0.069	0.061	0.067	0.040	0.020				
3	PH7	0.071	0.073	0.071	0.073	0.077	0.069	0.075	0.063	0.077	0.091			
4	PH3	0.048	0.046	0.048	0.051	0.050	0.046	0.048	0.074	0.084	0.098	0.095		
	PH6	0.068	0.066	0.068	0.076	0.069	0.070	0.072	0.070	0.063	0.081	0.093	0.029	
	PH8	0.056	0.062	0.056	0.066	0.058	0.062	0.060	0.070	0.061	0.075	0.100	0.036	0.018

**Figure 5.**
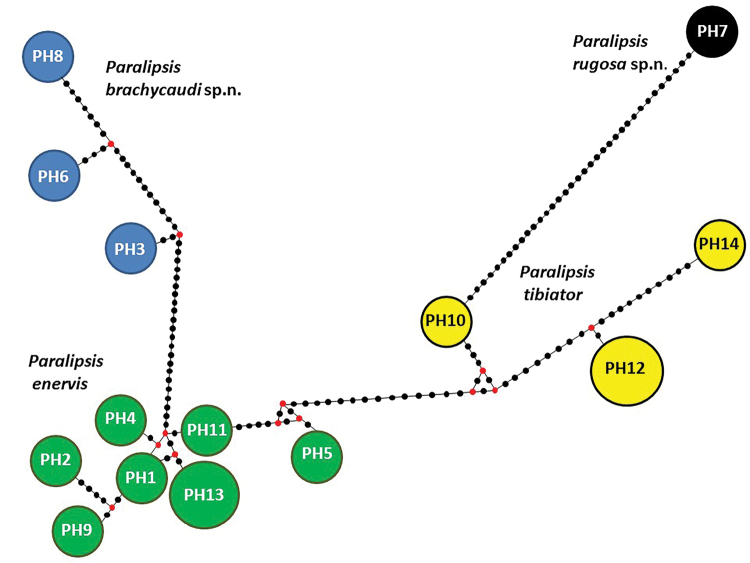
Median-joining network obtained for 14 *Paralipsis*COI barcoding haplotypes. Green circles represent group 1 (*P.enervis*), with haplotypes PH1, PH2, PH4, PH5, PH9, PH11, and PH13; yellow circles represent group 2 (*P.tibiator*), with haplotypes PH10, PH12, and PH14; the black circle represents the single haplotype PH7 from Moldova within group 3 (*P.rugosa* sp. n.); blue circles represent group 4 (*P.brachycaudi* sp. n.), consisting of haplotypes PH3, PH6, and PH8. Circle size reflects the number of individuals with that haplotype (not to scale). Red dots are median vectors. Black dots are mutational steps.

The median-joining network recognized the same four distinct groups of mitochondrial haplotypes with a confidence limit of 95%: group 1 (*P.enervis*) – haplotypes PH1, PH2, PH4, PH5, PH9, PH11, and PH13; group 2 (*P.tibiator*) – haplotypes PH10, PH12, and PH14; group 3 (*P.rugosa* sp. n.) – the single haplotype PH7 from Moldova; and group 4 (*P.brachycaudi* sp. n.) - haplotypes PH3, PH6, and PH8 (Figure 5). Using maximum parsimony calculation, we determined that all haplotypes are connected with no ambiguities, and the median vectors representing either unsampled or extinct haplotypes. A significant number of mutational steps (up to 40) connecting the groups confirms clear separation of the lineages, which corresponds with high divergence rates between the groups (group 1 and group 2 – 5.2%; group 1 and group 3 – 7.3%; group 1 and group 4 – 5.9%; group 2 and group 3 – 7.7%; group 2 and group 4 – 7.5%; and group 3 and group 4 – 9.6%).

### *Paralipsisenervis* – a review of host aphids and distribution of associations

The presented review includes evidence obtained for the most part from consulted published references about the species. The material was often re-visited, which was possible due to its preservation in available collections (IECR and FBS). The review also includes some new supplementary records (*).

Eriosomatinae

Pemphigini:

*Pemphigus* sp.: Czech Republic (Starý 2006).

Eriosomatini:

*Tetraneuraulmi* (L.): Czech Republic (Starý 1972, 2006), England (Pontin 1960), Germany (Starý 1961), Sweden (Hincks 1949).

Fordini:

*Fordaformicaria* von Heyden: England (Hincks 1958, Pontin 1960), Germany (Starý 1961), Serbia (Kavallieratos et al. 2004, Žikić et al. 2012), *Spain (Leon, 13.06.2013, leg. N Pérez Hidalgo).

*Fordamarginata* Koch: Lithuania - Molety, distr. Žičkai, 1-VIII-2012, on *Poaannua* roots, sample 12HAO4563 I male (J Havelka)

*Spain (Arcos de las Salinas, Teruel, 24/05/2017).

*Geoicautricularia* (Passerini): Serbia (Kavallieratos et al. 2004, Žikić et al. 2012).

Anoeciinae

*Anoeciacorni* (Fabricius): Germany (Völkl et al. 1996).

*Anoecia* sp.: England (Pontin 1960), Czech Republic (Starý 1961), France (Noury 1962).

Aphidinae

*Anuraphiscatonii* Hille Ris Lambers: Czech Republic (Starý 2006).

*Anuraphisfarfarae* (Koch): Czech Republic (Starý 2006), Slovakia (Starý and Lukáš 2009).

*Anuraphissubterranea* (Walker): England (Pontin 1960), Czech Republic (Starý 1961).

*Dysaphiscrataegi* (Kaltenbach): Czech Republic (Starý 1961, 2006).

*Dysaphisapiifoliapetroselini* (Börner): Spain (Suay Cano and Michelena Saval 1998).

*Dysaphisreaumuri* (Mordvilko): *France (La Combe, Hte. Savoie, 12.07.1989, *Ranunculus* sp., leg. G Remaudière).

*Brachycaudusballotae* (Passerini): Czech Republic (Starý 1961, 2006).

*Brachycauduscardui* (L.): Czech Republic (Starý 1961, 2006).

*Brachycaudusjakobi* Stroyan: Netherlands (van Achterberg and Ortiz de Zugasti Carrón 2016)

*Brachycaudusmordvilkoi* Hille Ris Lambers: Czech Republic (Starý 2006).

*Brachycaudus* sp.: Czech Republic (Starý 1961, 2006).

*Aphislambersi* (Börner): Slovakia (Starý and Lukáš 2009).

*Aphisroepkei* (Hille Ris Lambers): Czech Republic (Starý 2006).

*Aphisrumicis* L.: *Morocco (Immouzer, 28.04.1985, leg. A Sekkat).

*Protaphisterricola* Rondani: Russia-Western Siberia (Davidian and Gavrilyuk 2014).

This integrated review contains broad information and also allows a cross-comparison of all the known host aphid-parasitoid locations of *P.enervis* in the Western Palaearctic. The true distribution range of *P.enervis* is somewhat more extensive than that derivable from the above review, since in most of the countries the parasitoid wasp was determined from individually sampled specimens with no data on the associated host aphids. Similarly, the distribution data reflect strength of the respective field research efforts. It seems that the northern distribution limits are the Scandinavian countries. The vertical distribution also manifests some peculiarities. *Paralipsisenervis* was also reared from the root aphid *Dysaphisreaumuri* sampled in the Alps (France) at approximately 2200 meters (see the review).

### Descriptions of new species in Europe

On the basis of morphological examination of our available material from across Europe and the Mediterranean and using the COI mitochondrial barcoding marker, we confirmed the existence of the recently described *Paralipsis* species *P.tibiator*. In addition, two new *Paralipsis* species are described below.

#### Paralipsis brachycaudi

Taxon classificationAnimaliaHymenopteraBraconidae

Tomanović & Starý sp. n.

http://zoobank.org/E2918E28-9DA3-41C1-8ABA-3546CF423793

Figures 6–14

##### Material.

Holotype ♀, Czech Republic, Čejč, 28.V.1963, reared from *Brachycaudusmordvilkoi* Hille Ris Lambers on *Echiumvulgare* L., leg. J Holman; deposited in the IECR collection, slide mounted.

Paratypes 2♀♀, Czech Republic, Prague, 26.IX.1960, reared from *Brachycaudusballotae* (Passerini) on *Ballotanigra* L., leg. J Holman; deposited in the FBS collection, slide mounted. Germany, Erlangen, Nordbayern, reared from *Tetraneuraulmi* (L.) on *Avenasativa* L., leg. H. Zwölfer; deposited in the IECR collection, slide mounted.

##### Diagnosis.

The new species morphologically resembles *P.enervis* in petiole shape, absence of longitudinal placodes from flagellomeres 1 (F_1_) and 2 (F_2_), and fore wing venation pattern. *Paralipsisbrachycaudi* sp. n. differs from *P.enervis* in having a longer petiole (Figure 13), the ratio between petiole length and width at the spiracle level is 1.50-1.60 in *P.brachycaudi* sp. n., while in *P.enervis* it is 1.30–1.40; somewhat shorter F_1_ and F_2_ (Figure 7) (the ratio between length and width of F_1_ and F_2_ is approximately 2.00 in *P.brachycaudi* sp. n., as opposed to 2.20–2.30 in *P.enervis*); and a propodeum that is smooth with just a few rugosities at the side (Figure 9), while the propodeum in *P.enervis* sometimes possess rugosities in the central parts which indicate for the presence of a central areola. Additionally, F_1_ and F_2_ are light-brown to yellow in *P.brachycaudi* sp. n., while in *P.enervis* only half of flagellomere 1 is yellow and the remaining parts of the flagellomeres are brown. The ovipositor sheath in *P.brachycaudi* sp. n. (Figure 14) is more elongated than in *P.enervis*.

**Figures 6–14.**
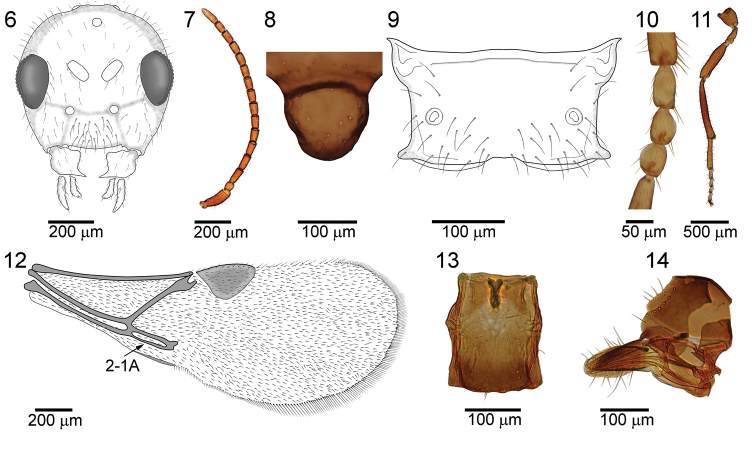
*Paralipsisbrachycaudi* sp. n., female **6** head, anterior view **7** antennae, lateral view **8** scutellum, dorsal view **9** propodeum, dorsal view **10** second-fourth segments of fore tarsus, dorsal view **11** hind leg, lateral view **12** fore wing **13** petiole, dorsal view **14** ovipositor sheath, lateral view.

##### Description.

***Female***: *Head* (Figure 6) rounded, narrower than mesosoma at tegulae, bearing sparse setae (Figure 6). Head 1.1 times wider than long medially. Eyes oval, small with scarse and long setae. Tentorial index approximately 0.95. Clypeus with 15–20 long setae. Maxillary and labial palpi with one palpomere each. Ocular-ocelar line: diameter of posterior ocellus: Postocelar line=12:4:14. Malar space: height of eye =20:26. Antenna 16-segmented, filiform (Figure 7). Scapus widened at the tip, vase shaped at lateral view. Pedicel subspherical. F_1_ equal to F_2_ and F_3_ and 2.0–2.1 times as long as its maximum width at the middle. Penultimate flagellomera 1.6 times as long as wide. F_1_, F_2_ and F_3_ without and F_4_ with one short longitudinal placode (Figure 7). Flagellomeres covered uniformly with short appressed and semi-erect setae.

*Mesosoma*: Mesoscutum smooth, and only moderately sculptured within small central area, usually with four rows of setae along its dorsolateral part. Mesoscutum 1.4 times as long as wide. Scutellum (Figure 8) smooth elongated, bearing 20–30 long setae in the central part. Propodeum (Figure 9) smooth, sometimes with rugosities laterally. Upper and lower parts of propodeum with 3–5 and 15–20 long setae on each side (Figure 9). Fore wing (Figure 12) densely pubescent, with long marginal setae, longer than those on fore wing surface. Vein 2-1A sclerotized (Figure 12). Pterostigma triangular, 1.7–1.9 times as long as its width (Figure 12). Secondfourth segments of fore tarsus in dorsal view (Figure 10) almost as long as wide (1.1–1.2 times as long as wide) and medium sized of apical bristles. Hind tibia medially and femur subbasally parallel-sided (Figure 11).

*Metasoma*: Petiole (Figure 13) smooth, with prominent spiracular tubercles, its length 1.50–1.60 times its width at spiracles and maximum width at level of spiracles 0.7 times distance between spiracle and apex of tergite 1; 10–15 setae positioned on posterior dorsolateral margin on each side. Ovipositor sheath (Figure 14) elongated, dorsally straight, narrowed toward tip, bearing 2–6 long setae on the ventral and dorsal surface. Length of ovipositor sheath 2.25–2.87 times its maximum width.

*Length*: body 1.5–2.0 mm; fore wing 1.3–1.7 mm.

*Coloration*: General body color light-brown to brown. Head brown with light-brown mouthparts. Scape and pedicel yellow to light-brown. Flagellomere 1 and 2 yellow, remaining parts of antennae brown. Mesosoma brown. Legs yellow to light-brown. Propodeum yellow. Metasoma brown. Petiole yellow. Ovipositor sheath dark-brown.

*Male*: unknown.

##### Etymology.

The name of the new species is derived from that of its aphid host.

##### Distribution.

Czech Republic, Germany.

#### Paralipsis rugosa

Taxon classificationAnimaliaHymenopteraBraconidae

Tomanović & Starý sp. n.

http://zoobank.org/8BA5B5C2-F16E-4006-AAC6-001662AC26B3

Figures 15–21

##### Material.

**Holotype** female, Moldova, Kišinev, 26.VI.1960, unknown aphid host and host plant, leg. Adaškevič; deposited in the IECR collection, slide mounted.

##### Diagnosis.

The new species differs clearly from all known *Paralipsis* species in having a strongly rugose propodeum (Figure 18) and scutellum (Figure 17) that are irregularly deep carinated, while other *Paralipsis* species are characterized by a smooth propodeum, sometimes with moderately expressed rugosities. Also, *P.rugosa* sp. n. is with F_1_ longer than F_2_ (the ratio between F_1_ and F_2_ is approximately 1.15) (Figure 16), while *P.enervis*, *P.brachycaudi* sp. n., and *P.planus* have F_1_ equal or subequal to F_2_. An exception is *P.tibiator*, which has much longer F_1_ than F_2_ (the ratio of F_1_ and F_2_ is about 1.4). Further, F_1_ and F_2_ are very short (proportion of length and width of F_1_ and F_2_ are 1.76 and 1.50, respectively) (Figure 16).

**Figures 15–21.**
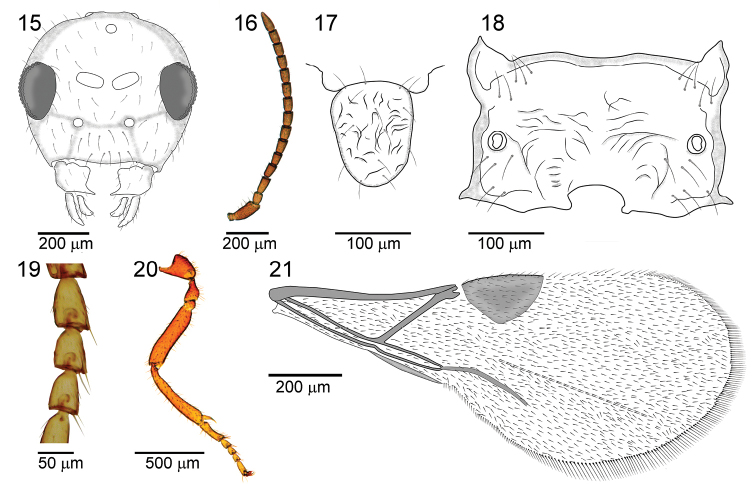
*Paralipsisrugosa* sp. n., female **15** head, anterior view **16** antennae, lateral view **17** scutellum, dorsal view **18** propodeum, dorsal view **19** second-fourth segments of fore tarsus, dorsal view **20** hind leg, lateral view **21** fore wing.

##### Description.

*Female*: Head rounded, smooth, narrower than mesosoma at tegulae, bearing dense setae (Figure 15). Head 1.1 times wider than long medially. Eyes oval, small, with scarse and long setae. Tentorial index 0.67. Clypeus with ten long setae (Figure 15). Maxillary and labial palpi with one palpomere each. Ocular-ocelar line: diameter of posterior ocellus: Postocelar line =12:4:14. Malar space: height of eye =11:13. Antenna 15-segmented, slightly thickened at apex (Figure 16). Scapus subapically with subparallel side at lateral view. Pedicel subspherical. F_1_ (Figure 16) longer than F_2_ and about 1.76 times as long as its maximum width at the middle, and F_2_ and F_3_ about 1.50 times as long as its maximum width at the middle. F_1_ and F_2_ without longitudinal placodes. Penultimate flagellomera 1.6 times as long as wide. F_1,_ F_2_ and F_3_ without, and F_4_ with one short longitudinal placode. Flagellomeres covered uniformly with short appressed and semi-erect setae (Figure 16).

*Mesosoma*: Mesoscutum smooth, sculptured laterally, with very dense setae laterally. Mesoscutum 1.4 times as long as wide. Scutellum subspherical, strongly rugose with about 15 setae (Figure 17). Propodeum (Figure 18) extremely rugose. Upper and lower parts of propodeum with 5–6 long setae on each side. Fore wing (Figure 21) densely pubescent, with long lower marginal setae, longer than those on fore wing surface. Pterostigma triangular, 1.62 times as long as its width. Vein 2-1A sclerotized. Metacarpus absent. Secondfourth segments (Figure 19) of fore tarsus in dorsal view distinctly longer than wide (1.4–1.8 times as long as wide) and medium sized of apical bristles. Hind tibia medially and femur subbasally parallel-sided (Figure 20).

*Metasoma*: damaged.

*Length*: head and mesosoma combined about 1 mm; fore wing about 1.7 mm.

*Coloration*: General body color brown. Head brown. Mouthparts light-brown. Scape and pedicel brown with small yellow terminal part. F_1_ and F_2_ yellow, remaining parts of antennae brown. Mesosoma brown to light-brown. Legs yellow to light-brown.

*Male*: unknown.

##### Etymology.

The name of the new species refers to the very rugose propodeum and scutellum.

##### Distribution.

Moldova.

### Key to the species of the genus *Paralipsis* Foerster on the basis of females

**Table:** 

1	Propodeum and scutellum with strong and deep rugosities (Figs 17, 18); F_1_ and F_2_ stout, 1.7 and 1.5 times as long as wide, respectively (Fig. 16); pterostigma triangular, about 1.6 times as long as wide (Fig. 21)	***P.rugosa* sp. n.**
–	Propodeum and scutellum smooth or with moderate expressed rugosities (Figs 8, 9); F_1_ and F_2_ elongate, 2.0–2.2 and 1.8–2.1 times as long as wide, respectively (Fig. 7); pterostigma 1.8–2.0 times as long as wide (Fig. 12)	**2**
2	Second-fourth segments of fore tarsus distinctly longer than wide in dorsal view (Fig. 22); hind tibia medially and femur subbasally widened (Fig. 23); flagellar segments narrowed basally	**3**
–	Second-fourth segments of fore tarsus approximately as long as wide in dorsal view (Fig. 19); hind tibia medially and femur subbasally almost parallel sided (Fig. 20); flagellar segments parallel-sided	**4**
3	Mesoscutum and scutellum smooth and densely setous; flagellomere 1 distinctly longer than F_2_ (1.3–1.4 times as long as wide); pterostigma triangular, approx. 1.8 times as long as wide	*** P. tibiator ***
–	Mesoscutum and scutellum moderate rugose and setous; F_1_ subequal to F_2_ (about 1.1 times as long as wide); pterostigma twice as long as wide	*** P. planus ***
4	Forewing 2-1A vein absent; Japan and Far East	*** P. eikoae ***
–	Forewing 2-1A vein present, partly or completely sclerotized; Europe	**5**
5	Petiole 1.50-1.60 times as long as wide at spiracles level; F_1_ about 2.0 times as long as wide; F_1_ and F_2_ yellow	***P.brachycaudi* sp. n.**
–	Petiole 1.30–1.40 times as long as wide at spiracles level; flagellomere 1, 2.0–2.2 times as long as wide; basal third of F_1_ yellow till light brown and remaining part F_1_ and whole F_2_ brown	*** P. enervis ***

**Figures 22, 23.**
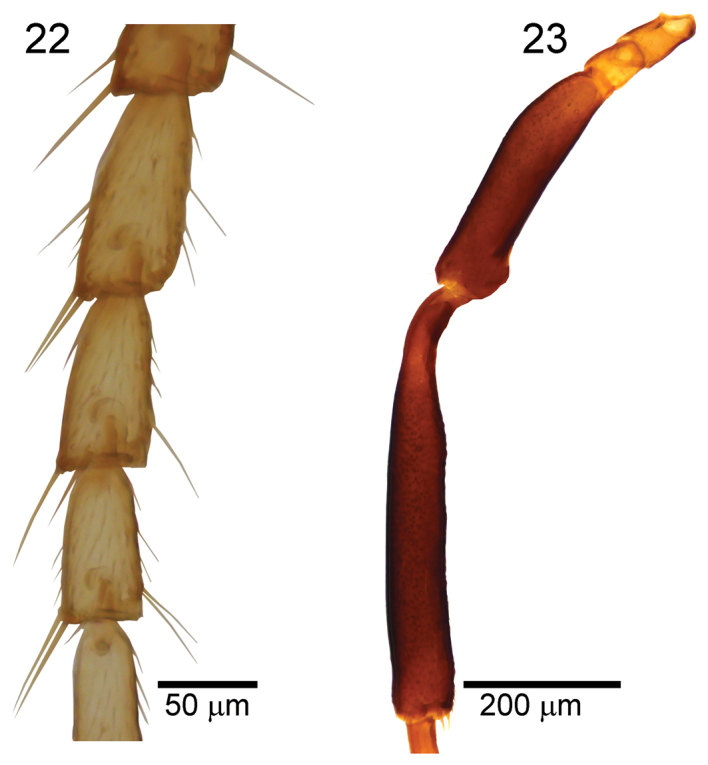
*Paralipsistibiator*, female **22** second-fourth segments of fore tarsus, dorsal view **23** hind tibia and femur, lateral view.

## Discussion

We have demonstrated here a progress in methodology of DNA amplification by designing *Paralipsis*-specific degenerative primers to retrieve disintegrated DNA fragments from archived museum specimen collections of which can be considered as biobanks and used to discover new species (Yeates et al. 2016). Sequencing of the COI barcoding region of 18 specimens collected across the Western Palaearctic over a long period of time did not determine any clear specialization of taxa to a strict root aphid host. There is no geographical structuring of genetic variation between specimens associated with the same aphid host within the lineages *P.enervis*, *P.tibiator*, nor *P.brachycaudi* sp. n. However, it confirmed the existence of *Paralipsistibiator*, a species recently described by van Achterberg and Ortiz de Zugasti Carrón (2016). Although a second recently described species, viz., *P.planus* (van Achterberg and Ortiz de Zugasti Carrón 2016), was not available for molecular analysis, the general morphological description (petiole shape, wing venation pattern, antennae) indicates that it is close to *P.enervis*, so we suppose it belongs to the *P.enervis* lineage. However, since *P.planus* was described on the basis of a single specimen, it is necessary to further explore the morphological and genetic variability of this species in order to finally confirm its taxonomic status. In addition, the present study revealed two new *Paralipsis* species, *P.rugosa* sp. n. and *P.brachycaudi* sp. n. Four separate phylogenetic lineages showed clear distinction, but with significant intralineage genetic variation between the haplotypes associated with different aphid/host associations. All phyletic lineages share aphid hosts from the subfamilies Aphidinae and Eriosomatinae. Many Eriosomatinae are specialized gall-producing aphids, but only on primary host plants, while this is not the case on secondary host plants (grasses), where they are parasitized by *Paralipsis* wasps and other specialized root aphid parasitoids.

It is necessary to examine in detail all known records of *P.enervis* in the light of the diagnosis given for the new *Paralipsis* species described in the present paper. Probably, *P.enervis* represents a complex of cryptic species, which is a common case among aphid parasitoids (Mitrovski-Bogdanović et al. 2013, Derocles et al. 2016). Although our molecular analyses were restricted to only 18 COI barcoding sequences retrieved from dry *Paralipsis* specimens, we recognized four phyletic lineages on the phylogenetic tree with a sister position of *P.brachycaudi* sp. n. in relation to *P.enervis* + *P.rugosa* sp. n. + *P.tibiator* lineages. The strong rugosities of the propodeum and scutellum in *P.rugosa* sp. n. represent an autapomorphic character state, while its possession of very short flagellomeres is a plesiomorphic character state. We recognize the elongated ovipositor sheath and petiole in *P.brachycaudi* sp. n. as apomorphic characters. We did not find any strong support for the existence of *Paralipsis* host-specific lineages. *Brachycaudus* aphid hosts were found only in the *P.brachycaudi* sp. n. lineage, while other aphid hosts are mainly shared between *P.enervis* and *P.tibiator*. Although *Forda* root aphids are distributed throughout the whole of Europe, *P.tibiator* attacked them only in Mediterranean-type habitats. It is known that the distribution of parasitoids is usually narrower than that of their aphid hosts, due to the more specific habitat and microhabitat of parasitoids (Starý 1970). The records of *P.enervis* associated with *Brachycaudus* aphid hosts should be carefully examined, as they may be referable to *P.brachycaudi* sp. n. All findings of *P.tibiator* are from Mediterranean areas.

Although most of our samples originated from central and southern Europe, *Paralipsis* species are distributed in several European countries, including ones in the northern part of the continent (van Achterberg 2012, Staverløkk and Ødegaard 2016). However, no *Paralipsis* species have been recorded in more than half of the countries of Europe (van Achterberg 2012). In the present study, we have not explored the relationships between ants and *Paralipsis* wasps. However, future research should reveal existing relationships of the two newly described species with ants and result in new knowledge about the biology and ecology of these *Paralipsis* wasps.

## Supplementary Material

XML Treatment for Paralipsis brachycaudi

XML Treatment for Paralipsis rugosa
